# Depolymerization of Polycotton‐Blended Fabrics: Challenges and Opportunities

**DOI:** 10.1002/cssc.202502002

**Published:** 2025-11-17

**Authors:** Elena Rosini, Jacopo La Rocca, Camilla Loro, Gianluigi Broggini, Loredano Pollegioni

**Affiliations:** ^1^ Department o Biotechnology and Life Sciences University of Insubria Varese Italy; ^2^ Department of Science and High Technology University of Insubria Como Italy

**Keywords:** circular economy, cotton, hydrolysis, polyethylene terephthalate, textile waste blends

## Abstract

The rapid growth of the textile industry has led to a dramatic increase in fiber production and textile waste, with blended fabrics—particularly made of polyethylene terephthalate (PET) and cotton—dominating global markets. These blends combine the advantageous properties of synthetic and natural fibers but pose significant challenges for recycling due to their composite structure and the required, often harsh, treatments. Traditional mechanical recycling falls short in effectively separating these materials, often resulting in downcycling or material loss. In recent years, innovative chemical and enzymatic recycling strategies have emerged, offering selective depolymerization routes to recover high‐purity monomers such as terephthalic acid and ethylene glycol from PET and glucose from cotton. These processes show promise for reintegration into polymer synthesis as well as valorization into bio‐based product streams. This minireview critically evaluates current approaches to cotton/PET hydrolysis processes, highlighting recent advancements in pretreatment techniques and process integration.

## Graphical abstract

The increasing production of cotton/polyethylene terephthalate (PET)‐blended textiles and the limitations of traditional recycling highlight the need for innovative strategies. This minireview explores pretreatments, chemical and enzymatic hydrolysis to selectively recover glucose, terephthalic acid, and ethylene glycol, enabling their reuse in recycled polymers and bio‐based products.

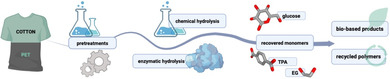

## 1 Introduction

In recent decades, textile production—especially for clothing—has increased exponentially, largely due to globalization. The global textile industry produces millions of tons of waste annually, with fiber production exceeding 111 million metric tons and projected to reach 146 million metric tons by 2030 [1, 2]. This growth is largely driven by the use of polyethylene terephthalate (PET) and cotton mixtures, often combined into polycotton‐blended fabrics that dominate applications across fashion, home furnishings, and industrial textiles. These blends combine the best properties of both materials, durability, wrinkle resistance, and hydrophobicity of PET, alongside the comfort and breathability of cotton. However, the heterogeneous composition of cotton/PET textiles poses a substantial challenge to efficient recycling and valorization [3, 4].

Traditional recycling approaches, primarily mechanical, are often inadequate for blended textiles due to the difficulty in separating synthetic and natural fibers, leading to downcycled or low‐value products, and thus contributing to soil pollution and greenhouse gas emissions. This has fueled growing interest in sustainable chemical and enzymatic recycling strategies capable of selectively depolymerizing either the PET or the cotton component to recover high‐purity monomers or sugars for repolymerization or bio‐based valorization [5–7]. On this side, achieving selective degradation while preserving the integrity of the nontargeted fiber remains a central scientific and technological challenge. Additionally, the presence of dyes and additives (aimed to enhance fabric performance) further complicates the recycling process, limiting the usability of recovered fibers [8].

Cotton fibers are primarily composed of crystalline cellulose, a polysaccharide polymer consisting of *β*‐1,4‐linked glucose units. The enzymatic hydrolysis of cellulose by cellulase enzymes is well‐established, producing glucose and oligosaccharides under mild aqueous conditions [9, 10]. This biocatalytic approach is attractive for selectively removing the cotton component from textile blends without affecting PET fibers. In order to optimize selectivity and efficiency, enzymatic treatment can be tailored by adjusting enzyme specificity, temperature, pH, and residence time. Recently, the enzymatic hydrolysis highlighted the almost complete recovery of glucose when applied to cotton/PET mixtures, without compromising PET depolymerization to terephthalic acid (TPA) [6, 7].

PET is a semicrystalline thermoplastic polyester extensively used in textile fibers and packaging materials. It can be chemically depolymerized by various methods, including hydrolysis (acidic or alkaline), glycolysis, methanolysis, and aminolysis, producing monomers such as TPA, bis(2‐hydroxyethyl) terephthalate (BHET), and ethylene glycol (EG). However, traditional chemical depolymerization often requires harsh reaction conditions, such as elevated temperatures, pressures, and strong acids or bases, which may damage and contaminate the cotton fraction in blends and add downstream processing complexity [11]. Recent advances in enzyme engineering, such as the development of PET‐degrading enzymes like PETase and cutinases, offer potential for direct enzymatic degradation of PET under environmentally sustainable conditions [12, 13–14]. Interestingly, a recently developed biotechnological process operates on polycotton fibers without melt‐amorphization, enabling the complete recovery of glucose and TPA [7].

This minireview aims to provide a comprehensive overview of recent advances in chemical and enzymatic depolymerization strategies of polycotton blended textiles, highlighting the critical role of pretreatment processes, which significantly influence the efficiency and selectivity of depolymerization. Several recent reviews have provided valuable overviews of textile recycling and depolymerization strategies, either focusing on single‐component systems—e.g., PET‐based fibers [15, 16] or cotton‐dominated blends [17]—or on specific processing routes, such as chemical depolymerization [15, 17] or physicochemical‐mechanical strategies [18]. More broad‐scope assessments have also appeared [16–19], generally covering multiple fiber classes and technologies at a high level. In contrast, this minireview is explicitly centered on real PET/cotton‐blended textiles and adopts a process‐driven, integration‐oriented perspective, uniquely combining (i) pretreatment strategies, (ii) selective chemical and enzymatic depolymerization workflows, and (iii) analytical benchmarking and pilot‐scale implementation considerations. This blend‐specific and application‐oriented approach fills a gap not covered by the existing literature. The presented strategies not only offer sustainable solutions but also contribute meaningfully to the development of a circular economy and the reduction of environmental impact.

## 2 Physico‐Chemical‐Mechanical Pretreatments

### 2.1 Decolorization and Removal of Additives

**FIGURE 1:**
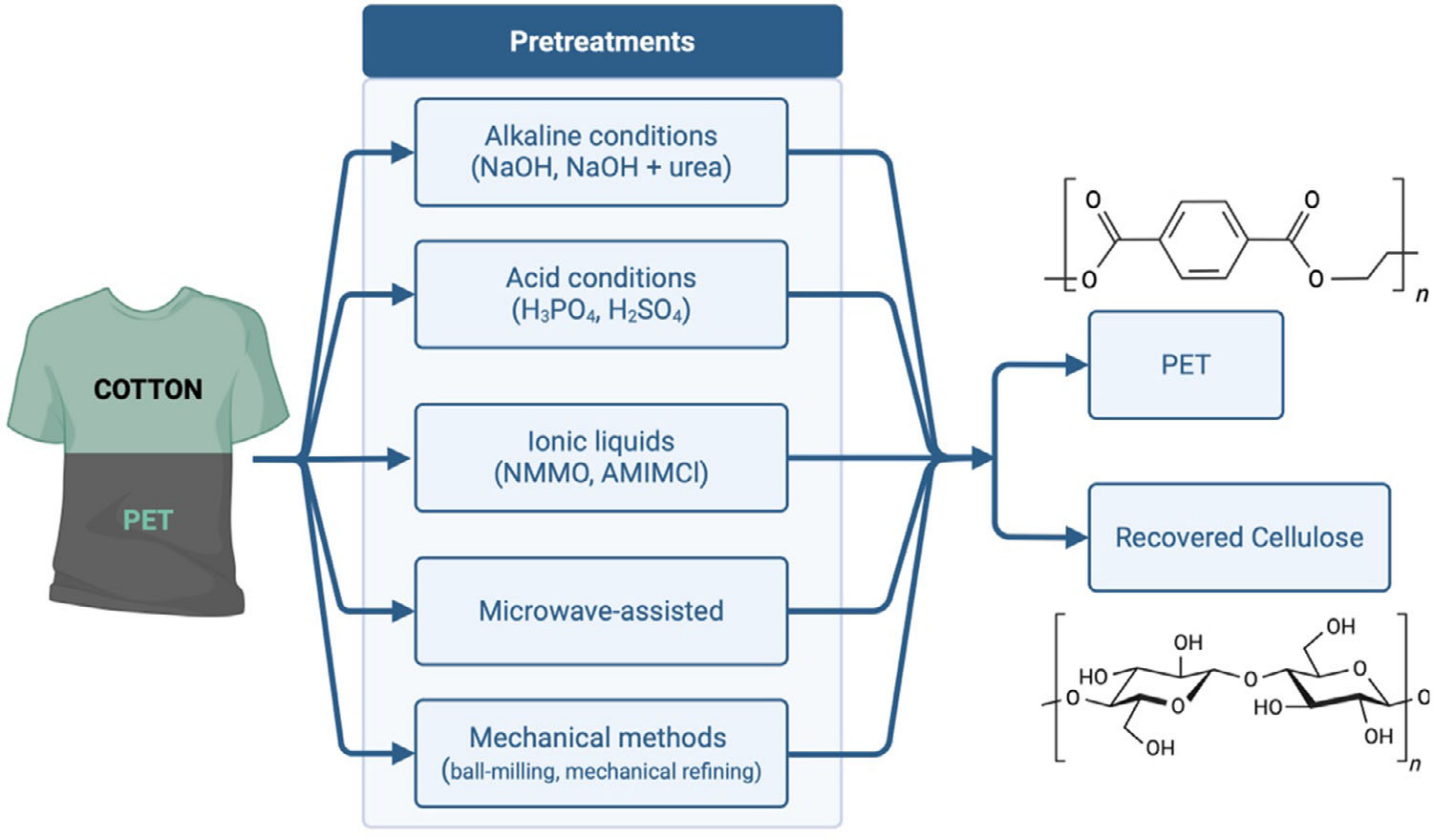
Overview of pretreatment strategies for cotton/PET‐blended textiles. Various approaches enable the separation and recovery of PET and cellulose components from textile fibers.

Pretreatments of textile materials are often applied to improve the efficiency of degradation processes. They are particularly useful for removing dyes and reducing the degree of crystallinity, see Figure 1.

The presence of dyes can represent a critical issue that negatively affects the purification process after chemical treatments, as well as the efficiency of enzymatic treatments. The dyeing of polycotton fabrics is achieved using reactive dyes applied to cotton fibers and/or disperse dyes applied to PET fibers. In the case of reactive dyes, bleaching treatments based on redox or high‐pressure alkaline processes can be used. Among the former, a recent approach is based on a two‐step process, where fabrics are treated with sodium dithionite and NaOH at 110°C for 90 min, followed by an oxidative reaction with hydrogen peroxide and NaOH at 90°C for 60 min [20]. On the other hand, among the alkaline treatments, it is worth mentioning the method proposed by Nahar and coworkers by heating at 121°C and 103.4 kPa, also used in a pilot plant, which allows complete decolorization of dyes combined with the separation of PET as TPA [21]. To remove dispersed dyes from PET, treatment with boiling EG vapor for 10–30 min is also effective [22].

### 2.2 Physico‐Chemical Pretreatments

Enzyme hydrolysis generally requires pretreatments aimed at reducing the crystallinity and ordered structure of textile fibers so as to improve their accessibility. Applying a pretreatment to cotton can increase the yield of enzymatic hydrolysis by up to 60–90% with respect to the direct treatment [6, 7, 23, 25–26]. Pretreatments of polycotton fabrics that have to undergo enzymatic degradation can follow different strategies, including alkaline or acid treatments, microwave‐assisted techniques, or the use of ionic liquids (Figure 1). Typically, these processes act on the cotton component of the fiber.

A significant example of alkaline pretreatment was reported by Boondaeng in 2023. Working with a 15% NaOH solution at 121°C for 15 min on textile waste blends of cotton and PET 35/65 or 60/40, the subsequent enzymatic treatment resulted in a complete cotton hydrolysis [10]. In addition, the combined use of 7% NaOH and 12% urea on polyester/cotton fiber blends resulted in 91–98% yields [4, 27, 28].

Acid treatments before enzymatic hydrolysis also showed good results, even if prolonged use of harsh conditions may cause drawbacks. In particular, strong acids can impair the effectiveness of the subsequent enzymatic conversion due to factors that hinder hydrolysis, such as cellulose degradation, reduced crystallinity, and increased flocculability [23]. The use of 85% phosphoric acid at 50°C for 7 h on a blend of polyester and cotton jeans resulted in 79% cotton degradation yield and complete recovery of the polyester component [23]. In 2019, Sasaki and coworkers developed an interesting pretreatment combining sulfuric acid with a microwave activation: the microwave irradiation at 200°C in the presence of 0.5% sulfuric acid applied to towels containing 88% cotton resulted in an enzymatic 80% degradation yield [29].

The use of ionic liquids has also shown valuable results in the pretreatment of enzymatic processes; however, their high cost represents a significant limitation to their application on an industrial scale, although they can be reused after the water has evaporated. In 2010, Jehanipour and coworkers pointed out a procedure using *N*‐methylmorpholine oxide monohydrate (NMMO) (85% w/w) at 120°C for 2 h for the conversion of mixed fabrics in glucose with a 90% yield [24]. Operationally, this method entails dissolving the fibers in an ionic liquid, followed by regeneration of the cellulose through the addition of water, filtration, and washing steps.

In enzymatic processes, in order to achieve satisfactory degradation of PET, the degree of crystallinity must be reduced from 30– 40% to 10%. In this case, the most effective pretreatment requires an extrusion process, followed by rapid cooling and subsequent micronisation. This allows PET transition from a semicrystalline state to an amorphous state, which is more accessible to enzymatic hydrolysis, after the necessary removal of the cellulose component [30, 31].

### 2.3 Mechanical Pretreatments

Conventional pretreatment methods may have some limitations, such as high costs, recovery issues, environmental concerns about wastewater, the need for corrosion‐resistant equipment, and elevated energy consumption. To address these drawbacks, mechanical approaches have been proposed [26]. Although they do not significantly reduce fiber crystallinity, they enhance the specific surface area, water retention capacity, and enzymatic adsorption, thereby facilitating hydrolysis [32]. For instance, Kaabel reported a 30% TPA recovery from PET after ball‐milling at 30 Hz on postconsumer cotton/PET 35/65 fibers [6]. Notably, in a recent patent, a stepwise ball‐milling and thermal pretreatment of a polycotton textile resulted in the near‐complete recovery of glucose, TPA, and EG [7]. However, a limitation of mechanical approaches arises from their inability to remove dyes or finishing agents present in postconsumer textiles, which can interfere with the subsequent enzymatic hydrolysis.

## 3 Chemical Degradation

Chemical recycling is a transformative process that depolymerizes cotton and PET polymers into their respective monomers or fundamental components. This approach is particularly effective for recycling fiber blends, as it allows for the selective depolymerization of targeted polymers within the mixture. The separation of individual components significantly increases the overall process efficiency. Furthermore, this method demonstrates itself to be advantageous in the presence of contaminants such as dyes, chemical additives, or metallic finishing agents, as it enables the recovery of high‐purity monomers, comparable in quality to their virgin counterparts.

### 3.1 Chemical Hydrolysis of Cotton

**FIGURE 2:**
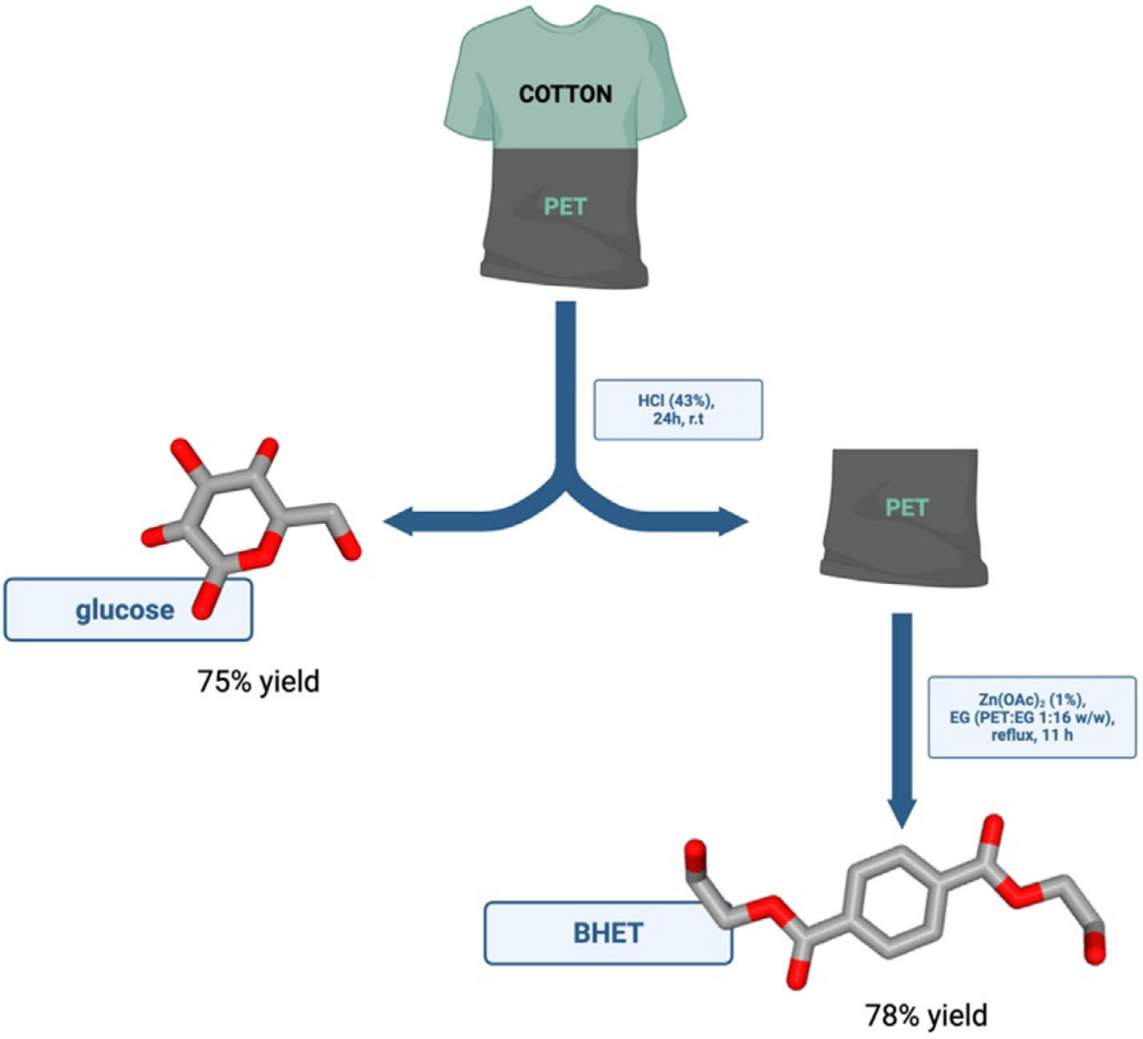
Overview of the treatment of polycotton fibers with hydrochloric acid.

In the case of cotton, chemical treatments typically rely on strong acids like formic and sulfuric acid in concentrated conditions. However, these approaches are characterized by low yields, and the use of high acid concentration and are confined to laboratory‐scale applications [33–35]. Using hydrochloric acid (1.5%) as a catalyst, cotton fibers were successfully solubilized in a polycotton fabric (65/35 cotton/PET) under hydrothermal conditions at 150°C for 3 h [36]. This treatment enabled the recovery of PET with a 96% yield, without significant alteration of its properties and achieved a 16% conversion of the cotton component into glucose. Hydrochloric acid was also employed in a recent study [37], where a superconcentrated solution (43%) was used to treat polycotton fibers, resulting in the complete dissolution of the cellulosic component and the 75% conversion of cotton to glucose, at room temperature in 24 h (Figure 2). The process was subsequently scaled up on a 230 L pilot plant. In an initial test, 11.8 kg of textile waste consisting of 100% cotton was processed without any pretreatment or agitation using a 43% aqueous HCl solution for 24 h: this resulted in complete solubilization of the cellulosic fraction and a glucose and cellobiose yield of 75%. In a second experiment, 26 kg of polycotton material (65% cotton) was subjected to the same treatment for 48 h, achieving a 75% glucose yield. In both cases, neither agitation nor mechanical pretreatment was employed, making additional physical preparation steps unnecessary.

### 3.2 Chemical Hydrolysis of PET

Conversely, the chemical recycling of PET is comparatively more optimized, benefiting from milder processing conditions, higher yields, and greater scalability. Extensive literature documents the alkaline hydrolysis of PET, particularly from bottle or packaging grade sources, often enhanced using phase‐transfer catalysts (PTCs), cosolvents [38], or microwave irradiation [39, 40]. These hydrolysis protocols have been extended to PET‐based textiles and blends [41]. Bottle and textile PET typically exhibit higher crystallinity (~30%) compared to PET packaging (~10%) [42]. However, pretreatment is not required for chemical hydrolysis, unlike enzymatic approaches, where substrate accessibility plays a critical role.

Laboratory‐scale experiments have demonstrated that textile PET can be efficiently hydrolyzed using alkaline conditions, typically at 90°C for durations ranging from 9 to 24 h. Alkaline hydrolysis of PET generates disodium TPA and EG, both of which are soluble in an aqueous environment. Subsequently, the addition of sulfuric acid allows protonation of the terephthalate salt, promoting precipitation of TPA, which can be separated by filtration and recovered in pure form.

Bengtsson and coworkers reported a complete degradation of PET and mixed PET/viscose fabrics (containing 30% PET), previously cut into pieces of about 1 × 1 cm, by alkaline hydrolysis in a 5% NaOH aqueous solution [43]. The samples were treated with a 1:100 solid‐to‐liquid ratio at 90°C. For PET‐only fabrics, hydrolysis was completed within 24 h, while for mixed PET/viscose fabrics, it took longer than 24 h to achieve complete dissolution of the PET components. TPA was subsequently recovered by precipitation with 4 M H_2_SO_4_ and separation by filtration: the PET conversion into TPA was close to 100%.

Benzyltributylammonium chloride (BTBAC) is known as an efficient PTC for PET degradation reactions. Its cationic component facilitates the transport of hydroxide ions to the PET surface, enhancing nucleophilic attack and accelerating the depolymerization process [44]. In details, 5 g of ground polycotton fabric was treated with 500 g of an aqueous solution containing 10% NaOH and BTBAC at a concentration of 1 mol per mol of repeating PET unit (52 mmol/kg hydrolysis solution). Under these conditions, the PET component was completely degraded, and the cotton fibers were fully recovered, confirming the selective depolymerization of PET in blended textiles [45]. A separate study used a similar BTBAC loading (1 mol per mol of PET unit, equivalent to 1.7%) and a longer reaction time (270 min) at 80°C; this method yielded 80% TPA recovery from postconsumer cotton/PET denim waste [46]. Neither investigation evaluated the possibility of recovering or reusing the catalyst.

### 3.3 Scale‐Up and Integrated Recycling Processes

Gr3n Company [https://gr3n‐recycling.com/] developed a continuous microwave reactor capable of processing from 10 to 60 kg/h of inlet PET. Polycotton fabrics were ground and mixed with a solution of alkaline hydroxide and EG: the depolymerization reaction took approximately 10 min. Following treatment, TPA was recovered from the solution and crystallized, while EG was separated and purified through distillation. After TPA removal by HCl addition, the remaining aqueous acidic solution was neutralized with alkaline bases. The resulting salts were used in an electrolysis unit for a chlor‐alkali process, enabling the recovery of NaOH and HCl. The recovered NaOH was reused both as a catalyst for the depolymerization reaction and for neutralizing acidic byproducts, while the recovered HCl was employed to precipitate purified TPA from the solution: the recovered TPA was reacted with EG to synthesize regenerated PET [47].

**FIGURE 3:**
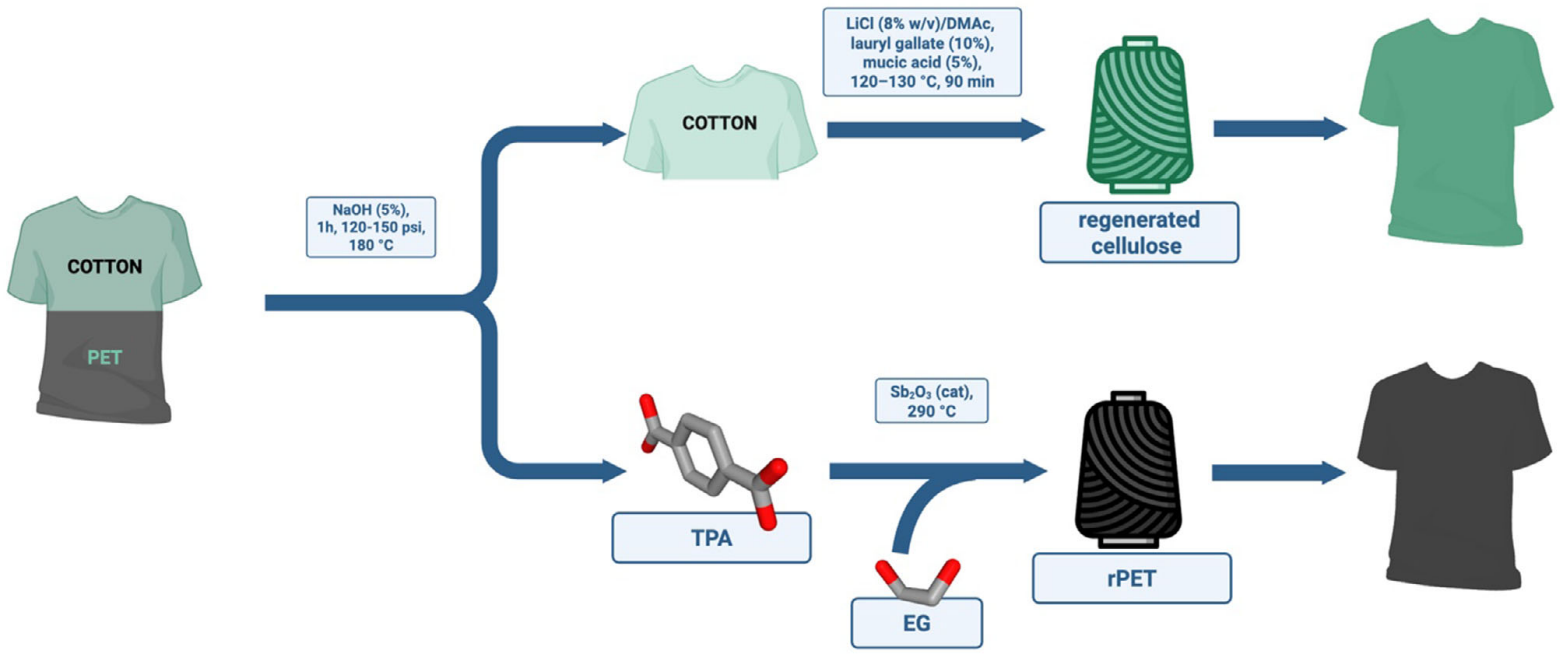
Recycling pathway of polycotton waste textiles in the Circ process using subcritical water treatment: the polyester fraction is hydrolyzed under alkaline conditions to yield TPA, then repolymerized with EG and Sb_2_O_3_ to form regenerated PET. Meanwhile, the resulting cellulosic pulp is activated, dissolved in LiCl/DMAc with additives, and spun into regenerated cellulose filaments for textile use.

Circ company (ex Tyton Biosciences) [https://circ.earth/] uses subcritical water to degradate polycotton blends (Figure 3): waste textile sheets were cut into approximately 30 × 30 cm pieces, introduced into a 2‐gallon hydrothermal reactor for subcritical water treatment, maintained at 180°C, with 5% sodium hydroxide solution (w/w) for 60 min, at a pressure ranging from 120 to 150 psi. The solid/water ratio was 1:11. After the treatment, the dissolved TPA was recovered from the solution and then reacted with EG to regenerate PET. The cellulose pulp was converted into a regenerated cellulose fiber produced via solvent‐spinning (Figure 3) [48].

The most significant results concerning the chemical PET degradation of polycotton are summarized in Table 1.

**TABLE 1:** Overview of the conditions for PET degradation by chemical methods.

Application scale	Material	Conditions	TPA Yield (%)	Ref.
Lab scale (5 g)	Viscose/PET (70/30)	5% NaOH, 90°C, >24 h	100	[43]
Lab scale (5 g)	Cotton/PET	10% NaOH, 1.8% BTBAC, 90°C, 40 min	100	[45]
Lab scale (0.1g)	Post‐consumer Cotton/PET (60–80 PET)	15% NaOH, 1.8% BTBAC, 80°C, 270 min	80	[46]
Lab scale	Cotton/PET (30/70)	NaOH, 121°C, 103.4 kPa, 90 min	98	[21]
Lab scale (4 g)	Cotton/PET (50–85 PET)	2% Zn(OAc)_2_, 210°C, 4 h	71–89	[22]
Pilot plant	Cotton/PET (PET > 70%)	NaOH, microwave, 10 min	100	[47]
Pilot plant	Cotton/PET	5% NaOH, 180°C, 120–150 psi, 60 min	100	[48]

## 4 Enzymatic Degradation of Blended Textile Waste

In response to growing environmental concerns and textile waste accumulation, innovative and sustainable enzymatic, green approaches to recover valuable building blocks from mixed textile blends have been recently proposed [8–10, 26]. The goal is to align with circular economy principles by transforming waste into reusable resources, instead of resorting to landfilling or incineration. Cotton–PET blends are among the most commonly used materials in textile manufacturing [49]. Combining PET with cotton enhances the fabric's properties while posing significant challenges in recycling. Actually, the shorter fiber length in cotton–PET mixtures leads to lower‐quality and weaker recycled fibers, making them unsuitable for reuse in clothing manufacturing. Additionally, the tightly woven structure of cotton and PET fabrics hinders effective enzymatic breakdown [26, 32]. On a broader scale, textile recycling is further complicated by difficulties in sorting and limitations in current recycling technologies. As a result, finding efficient methods to recycle cotton–PET blends remains a critical and actual area of research [50, 51].

### 4.1 Selective Enzymatic Hydrolysis of Cotton

Because of the high cellulose content, cotton‐rich textile waste is increasingly explored as a renewable energy source, particularly for bioethanol [24, 24, 27, 52] and biogas production [3, 24]. In these bioprocesses, enzymes like cellulases (see below) catalyze the breakdown of cellulose with release of glucose. Cotton's high crystallinity and polymerization—due to inter‐ and intra‐molecular bonds—make enzymatic degradation challenging [53]. Current research mainly focuses on pretreatment techniques aimed to facilitate access of enzymes to the internal structure of cellulose to maximize glucose recovery and preserve polyester. This purified PET can be reprocessed into granules and, based on its quality, either reused directly or further treated to achieve the appropriate viscosity for spinning. After that, it can be transformed into fibers and then spun into yarns.

**FIGURE 4:**
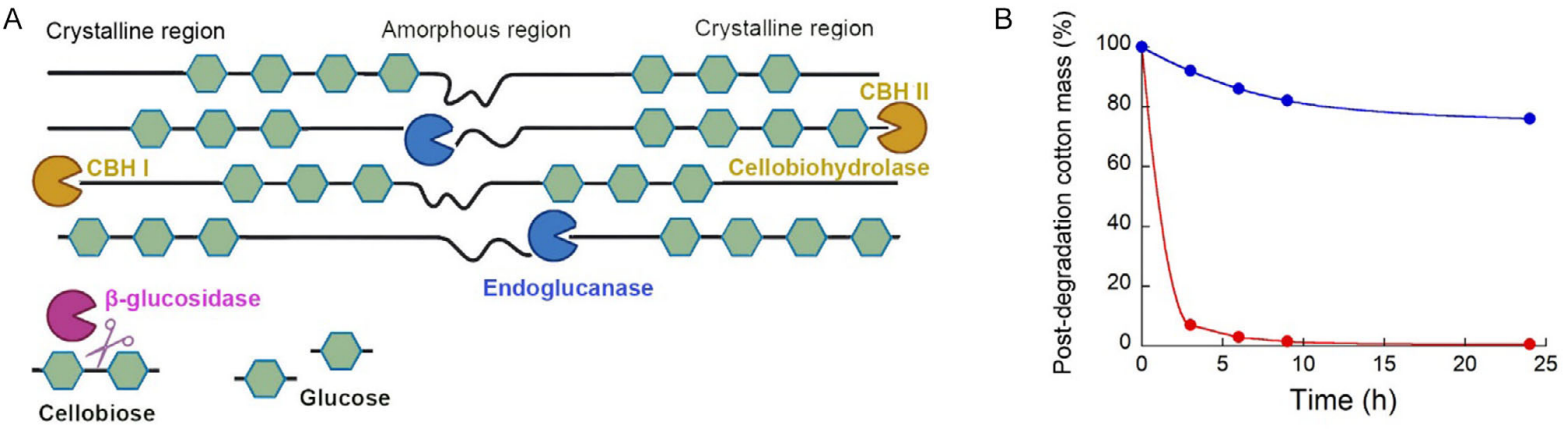
(A) Synergistic action of cellulases: endoglucanases (blue) cut internal *β*−1,4 bonds to generate new chain ends; cellobiohydrolases (CBH, yellow) release cellobiose from reducing (CBHI) and nonreducing (CBHII) ends; *β*‐glucosidases (pink) convert cellobiose and short oligosaccharides (≤6 monomers) into glucose. (B) Residual cotton mass after cellulase enzymatic degradation of untreated (blue line) and 20% NaOH‐treated (red line) fibers. Adapted from [51].

Cellulases are employed to remove the cotton material. These enzymes are readily available on the market and have been fine‐tuned for optimal performance by the biorefinery industry, where they are widely employed. Cellulases include several subclasses of enzymes with different functions: endoglucanases hydrolyze internal glycosidic bonds along the cellulose chain, cellobiohydrolases trim cellobiose units from the ends of the polymer, and *β*‐glucosidases hydrolyze cellobiose units into two molecules of glucose (Figure 4A) [54].

The effect of substrate loading, temperature, pH, and cellulases dosage on the hydrolysis process has been investigated, as well as different pretreatments for improving glucose recovery efficiency [4]. A blended textile of cotton and PET by 60/40 was subjected to two different pretreatments: i) freezing NaOH/urea and ii) milling. A significant enhancement of the glucose recovery was observed following the freezing NaOH/urea pretreatment: a value of 98.3% was achieved in comparison to 60.6% after the milling process. Glucose recovery strongly depends on the substrate‐to‐liquid ratio. Additionally, the pH value represents a fundamental physical parameter affecting the cellulase absorption to textile [55]. The highest glucose recovery was obtained using 20 FPU/g cellulase (one filter paper unit ‐ PFU ‐ is the amount of enzyme producing 2 mg of reducing sugars from 50 mg of filter paper in 1 h) and 10 U/g *β*‐glucosidase, at 3% (w/v) substrate loading, 50°C, and pH 5.0 [4]. Later on, a laboratory‐scale process to separate complex blends of 50/50 cotton/PET into different fiber fractions has been optimized [50]. In detail, fabrics were at first cut into 1 cm^2^ squares, and then added of cellulase solution (20:1 liquid‐to‐solid ratio) and transferred into a bottle containing PTFE stir bars, then incubated on a magnetic stirred temperature‐controlled block at 50°C for 19 h. After two sequential enzymatic treatments (at 5 and 10 FPU/g cellulase solution), almost complete separation of cotton from PET was achieved.

The process was then scaled up in a 54 L bioreactor to treat up to 50 kg of the cotton/PET textile [51]. To enhance enzymatic hydrolysis, the surface contact was increased by grinding the raw textile material in a cutting mill using a trapezoid screen (0.5 mm sized). The most effective pretreatment was based on soaking in 20% NaOH at room temperature for 1 h, followed by rinsing to neutral pH. A comparison between treated and untreated fibers is shown in Figure 4B. Enzyme concentration was also optimized, with 99% fiber degradation achieved at 1 g/L of cellulase solution. The procedure was successfully scaled up, enabling the hydrolysis of 3 kg of textile per batch and resulting in the overall production of approximately 30 kg of pure PET, sufficient for spinning trials and new textile development.

The effect of different concentrations of sodium hydroxide (10–30%) and urea (0–12%), as well as temperature in the −20 to 50°C range, during the pretreatment was investigated using a design of experiments approach [56]. Interestingly, cellulose undergoes a structural modification from its native crystalline form (cellulose I) into an intermediate alkali cellulose when treated with sodium hydroxide, and eventually to a more enzyme‐accessible form known as cellulose II. At low NaOH concentrations, cellulose fibers begin to swell: as the concentration increases, hydrogen bonds within the cellulose structure break, thus improving enzyme accessibility. When concentrations are raised further, a process called mercerization takes place, which converts cellulose I into cellulose II [57]. Moreover, several studies found that temperature increase promotes the shift from crystalline cellulose I to amorphous cellulose II. In contrast, other investigations suggest that lower temperatures, especially when combined with urea, can also enhance this transformation [49, 58, 59]. A factorial composite design was employed to investigate the interplay effects of urea and temperature on NaOH pretreatment of blended textiles (65% PET and 35% cotton), at moderate temperature values (selected to ensure economic feasibility). The model suggests that PET degradation is influenced by both temperature and NaOH concentration. At around –10°C, significant degradation occurs with below 15% NaOH levels. In contrast, temperatures above 40°C enhance degradation when NaOH concentration exceeds 25%. Additionally, higher urea levels amplify this effect, leading to mass losses of up to 36.4% at 50°C, 30% NaOH, and 12% urea. Notably, effective cellulose removal from the blended textile without compromising the PET structure, as judged by differential scanning calorimetry (DSC) and Fourier transform infrared spectroscopy (FTIR) analyses, was achieved at temperature values in the 6.2–13.3°C range, using either high NaOH concentrations (20.7–26.6%) without urea or a mix of 13.9% NaOH and 12% urea.

A life cycle assessment analysis of a process for the recovery of PET fibers and glucose from a 50/50 cotton/PET blended textile sample highlights alkaline pretreatment as the most impactful process, with melt spinning and enzymatic hydrolysis (using cellulases and *β*‐glucosidases) contributing less significantly [60]. Using 1 kg of recovered PET fibers as functional unit, pretreatment is the most energy‐intensive step (207 MJ), followed by melt‐spinning (98.5 MJ) and enzymatic hydrolysis (44.8 MJ). To prepare the textile waste for hydrolysis, the fabric was first shredded into small pieces using a double‐shaft shredder, which increases the surface area for the pretreatment [4]. The subsequent step involved mixing the shredded textile with a solution containing 7% NaOH and 12% urea at a solid loading of 5% (w/v) and cooling it to −20°C for 6 h. To purify the enzymatic hydrolysate from chemicals, activated carbon was added (15% w/w). The hydrolysate then underwent an ion exchange column to eliminate any remaining ions. The recovered glucose‐rich syrup can be used to produce bioplastics, biosurfactants, and biochemicals, whereas the polyester recovered from hydrolysis was respun into PET.

**FIGURE 5:**
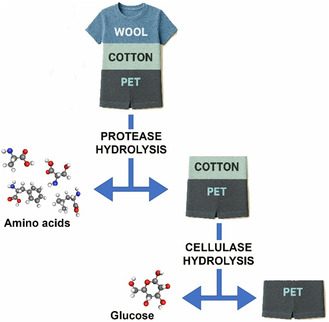
Stepwise degradation of mixed‐composition textile fibers (wool, cotton, and PET) via successive treatments with proteases and cellulases, enabling selective recovery of amino acids and glucose while leaving the PET fraction intact.

In view of an upcycling process to valorize building blocks recovered enzymatically from blended textile waste samples, a stepwise treatment was optimized [5]. The blended textile waste was sequentially treated with i) proteases, to extract amino acids from wool, and ii) cellulases, to recover glucose from cotton. This process is highly selective: while the enzymes break down wool and cotton, they leave the polyester fraction intact. A schematic representation of the stepwise process highlighting the enzymatically recovery of different components from blended materials, like cotton–polyester, wool–polyester, cotton–wool, or wool–cotton–polyester, is shown in Figure 5.

In detail, the samples were ground to 1 mm in size to enhance the available surface area for enzymatic treatment and improve mass transfer [61]. The artificial blends were then washed with mQ‐H_2_O and boiled for 30 min, followed by drying at 105°C for 6 h. Afterward, the samples were vacuum‐filtered, washed again in mQ‐H_2_O, and stirred at 400 rpm for 30 min, then filtered, and dried at 105°C for 6 h. Afterward, the sample was weighed and 1 g incubated in 50 mM Tris‐HCl buffer, pH 9.0, with 8 U/mL commercial protease (ratio with solid matter) for 2 days at 50°C, under magnetic stirring at 400 rpm. Next, the sample was incubated for 5 days at 50°C in 50 mM sodium citrate buffer, pH 4.8, containing a commercial cellulase cocktail (2750 U/mL) while stirring at 400 rpm. After hydrolysis, the sample was filtered and dried. A higher glucose recovery yield was observed for samples with higher cellulose content (90/5/5 and 80/10/10, cotton/wool/PET), a figure that decreased to 50% when the polyester increased to 40% (50/10/40, cotton/wool/PET). However, almost 80% glucose was recovered from real textile waste after cellulase treatment. For all tested samples, the enzymatic solubilization of wool resulted in a 95% recovery yield of amino acids. The purity of the remaining PET was >90% as verified by FTIR analysis. Noteworthy, the glucose obtained from cotton hydrolysis was effectively converted into ethanol through fermentation using the yeast *Saccharomyces cerevisiae*: after 6 h of fermentation, the maximum production of ethanol (≈0.2 g per liter) was obtained.

### 4.2 Enzymatic PET Depolymerization

In recent years, a number of natural and engineered PET hydrolyzing enzymes have been discovered [62, 63, 64, 65, 66, 67, 68–69] as comprehensively summarized by Rosini et al. [14] This opening to the green biodegradation of PET‐containing materials under mild conditions, normally in water and at 55–75°C.

Recently, mixed cotton/PET textiles have been directly and selectively depolymerized into glucose and TPA through simultaneous or sequential treatment with the cellulase CTec2 and the cutinase from *Humicola insolens* (HiC), both commercially available, under moist‐solid reaction conditions [6]. An innovative periodic mechanical agitation by ball‐milling, named reactive aging (RAging) [70, 71] was employed to improve the rate of enzymatic hydrolysis. Each RAging cycle consisted of 5 min of milling and 24 h of aging at 55°C. A sequential enzyme addition allowed to optimize the hydrolysis of a cotton/PET 35/65 textile blend. After an initial incubation with cellulase (yielding 68.6 ± 0.2% glucose recovery), the remaining material was dried and RAging resumed with the HiC enzyme. This approach achieved a 32% TPA yield and an overall weight loss of 62 ± 0.4% after five rounds, even on high crystalline PET samples (≥40%).

Finally, blended cotton/PET textiles were converted into value‐added chemicals in a biorefinery approach via enzymatic hydrolysis and fermentation [72]. Blended fabrics showed efficient cotton hydrolysis after chemical PET removal (using a 15% NaOH and 60% ethanol pretreatment, at 80°C for 2 h), resulting in a 92.5% glucose recovery yield. The recovered sugars were further converted into key bio‐based chemicals using the cellulase from *Trichoderma reesei* RUT C30 (20 FPU per gram of substrate) through fermentation or hydrogenation: glucose was selectively converted into sorbitol and lactic acid with yields of 70% and 83.7%, respectively, or fermented, yielding 537 mL of ethanol/kg textile. Additionally, the NaOH‐ethanol pretreatment achieved 86.5% PET removal and a 56.7% solid recovery. The following incubation of the PET fraction with the recombinant WCCG PET hydrolase, a leaf branch compost cutinase variant [12], resulted in depolymerization into the TPA and EG monomers, which are suitable for repolymerization into high‐value PET materials.

Very recently, an innovative, green biorefinery approach has been patented to valorize blended textile waste, particularly polycotton (70% PET, and 30% cotton) [7]. Physico‐mechanical pretreatments, combined with a stepwise enzymatic treatment using a mixture of commercial cellulases and the engineered cutinase S101N/F243T‐ΔLCC variant, enabled the selective production of glucose from cotton and the near‐complete hydrolysis of PET into TPA and EG.

### 4.3 Biorefinery and Upcycling Perspectives

Noteworthy, TPA is an aromatic building block with broad potential in sustainable upcycling. Through the canonical TPA dioxygenase pathway, TPA is converted into protocatechuic acid (PCA) and funneled into the *β*‐ketoadipate pathway for ring cleavage and assimilation [73]. Engineered microbial systems have greatly expanded its valorization routes. *Pseudomonas putida*, *Comamonas testosteroni*, and *Rhodococcus jostii* have been engineered to produce high‐value compounds such as muconic acid, *β*‐ketoadipic acid, vanillin, and lycopene [74–76]. Cocultivation strategies enable direct PET oligomer‐to‐PHA conversion, achieving yields up to 1.1 g/L PHA with tailored monomer composition [77]. TPA can also serve as a precursor for novel materials, e.g., microbial production of hydroxyalkanoyloxy‐alkanoates, followed by chemical polymerization yields bio‐based poly(amide urethane) with tunable thermal properties [78]. Whole‐cell biocatalytic systems have reached PET‐to‐muconic acid conversion rates of 0.5 g/g PET (68% theoretical yield) without intermediate accumulation [75]. These biotechnological advances position TPA as a versatile feedstock for commodity chemicals, functional monomers, and specialty products, integrating plastic waste valorization into a circular and bio‐based economy.

## 5 Characterization of Polycotton Fibers and their Degradation Products

The characterization of polycotton fibers, both in their native state and after chemical or enzymatic treatments, requires a multidisciplinary analytical approach: the integration of morphological, spectroscopic, thermal, mechanical, and chromatographic methods enables a deeper understanding of the treated materials (see Table 2).

Morphological variations before and after degradation treatments are typically evaluated using scanning electron microscopy (SEM), which reveals surface features like fibrillation, micropitting, or fiber thinning, indicators of partial hydrolysis and surface erosion [79, 80]. This method is particularly powerful for visualizing localized degradation phenomena and verifying the disappearance of PET or cotton fibers, thus confirming the selectivity and efficiency of degradation processes [23, 81, 32]. Nevertheless, SEM remains essentially qualitative: it provides no direct information on chemical composition or crystallinity, and sample preparation can introduce artifacts such as charging or surface damage. Complementary to SEM, optical microscopy can be employed to monitor macroscale surface smoothness or delamination effects, though its lower resolution limits the detection of early‐stage or nanoscale degradation [80]. At the molecular level, FTIR is a useful method to detect modifications in functional groups (e.g., hydroxyl, carbonyl, ester bonds). In polycotton fibers, FTIR spectroscopy equipped with a single‐bounce diamond attenuated total reflectance (ATR) is commonly used to identify PET degradation products, such as BHET and TPA, which are obtained by precipitation after glycolysis and hydrolysis [40]. The characteristic carbonyl bands, between 1713 and 1721 cm^–1^, and variations in the peaks associated with –OH groups, around 3331 cm^–1^, allow monitoring the process efficiency and assessing the cotton/PET blend composition [56]. Additionally, this technique enables the analysis of crystallinity variations in PET fibers after thermal treatments and in cotton fibers following alkaline treatments. In PET samples, peaks at 973 cm^–1^, 1340 cm^–1^, and 1473 cm^–1^ indicate the crystalline phase, while those at 898 cm^–1^, 1370 cm^–1^, and 1455 cm^–1^ correspond to the amorphous phase [82, 83–84]. In the case of cotton, bands between 4000 and 2995 cm^–1^, as well as those at 2900 cm^–1^, 1430 cm^–1^, 1375 cm^–1^, and 900 cm^–1^, are particularly sensitive to both crystalline and amorphous structures [85].

FTIR is semiquantitative and surface‐sensitive, which may lead to discrepancies between surface and bulk composition, an important distinction since enzymatic degradation of PET is an interfacial process [86].

Conversely, DSC provides bulk information. Parameters such as the glass transition temperature (T_g_), cold crystallization temperature (T_c_), and melting point (T_m_) are routinely used to evaluate overall crystallinity, a key factor affecting pretreatment and degradation efficiency [87]. However, the bulk T_g_ of PET is typically lower than the surface T_g_, further complicating the interpretation of DSC results when correlating them with enzymatic accessibility or degradation kinetics [86].

Quantitative analysis of hydrolysis products is fundamental. High‐performance liquid chromatography (HPLC) is widely used to quantify glucose released from cellulose: a ionic exchange column maintained at 50°C is used, with a 5 mM sulfuric acid‐based eluent [4, 34]. Regarding the analysis of PET degradation products, such as TPA, BHET, and MHET, elution of a C18 column is performed using a methanol gradient varying from 30% to 90% in 1 mM sulfuric acid [30, 88]. The identity of PET degradation products can be confirmed by ^1^H NMR, using DMSO‐d_6_ as the solvent [89]. Moreover, this technique allows for the determination of molecular weight and the quantification of hydroxyl and carboxyl groups through the use of solvents such as CDCl_3_ mixed with HFIP or TFA [90, 91].

**TABLE 2:** Comparative summary of analytical techniques for characterizing polycotton fibers.

Parameter	Analytical technique	Information provided	Posttreatment relevance
Surface morphology	SEM	Fiber damage, fibrillation, surface erosion	Chemical and enzymatic surface degradation
Macrosurface appearance	Optical microscopy	Macroscale changes (smoothness, delamination)	Assess polishing, bulk uniformity
Functional groups	FTIR‐ATR	Cellulose and ester bonds, OH, C =O, glycosidic/ester linkages	Cleavage mechanisms
Thermal properties	DSC	T_g_ and T_m_ of polyester, thermal stability	Polymer degradation and chain scission
Released sugars	HPLC, DNS assay	Glucose from cellulose hydrolysis	Quantify cellulase activity and efficiency
PET hydrolysis	HPLC	EG, TPA, BHET quantification	Ester bond cleavage

## 6 Summary and Outlook

The increasing consumption of polycotton blended textiles poses a significant environmental challenge due to the difficulty of recycling their composite natural/synthetic structure. Traditional mechanical methods are inadequate for the selective recovery of single components like glucose and TPA, prompting the development of more sustainable chemical and enzymatic depolymerization strategies.

This minireview has critically analyzed current methodologies, highlighting the pivotal role of pretreatments—mechanical, chemical, or physico‐chemical—in enhancing depolymerization efficiency. Chemical routes such as alkaline hydrolysis, often aided by PTCs or microwave irradiation, have demonstrated high TPA recovery from PET without affecting cotton. Conversely, enzymatic approaches allow for selective cellulose degradation and high glucose yields while preserving PET fibers.

These technologies show great promise in addressing the complex challenge of polycotton waste valorization. Through a combination of chemical and enzymatic processes, the selective recovery of high‐purity monomers and fermentable sugars from blended fabrics is feasible. In this context, continuous‐flow reactors and enzymatic bioreactors could represent a concrete step toward scalable implementation, offering a tangible bridge between laboratory innovation and real‐world industrial application.

Nonetheless, several technical and logistical challenges must still be addressed before large‐scale implementation becomes viable. One critical barrier is the effective removal of dyes, resins, and finishing agents, which can inhibit both enzymatic activity and product purity. Economic scalability remains another key issue, particularly regarding enzyme production costs, catalyst reuse, and reactor energy efficiency; all these aspects need to be carefully evaluated through life‐cycle and technoeconomic analyses. Beyond process chemistry, the success of these technologies also depends on upstream factors: efficient sorting of heterogeneous textile waste, traceability of fabric composition, and standardization of input materials will be essential to ensure consistent performance, yield, and product quality across industrial applications.

Looking forward, the integration of pretreatment, selective depolymerization, and upcycling into closed‐loop processes represents a promising avenue for transforming textile wastes into valuable resources. Multidisciplinary efforts combining enzyme engineering, materials science, process optimization, and life‐cycle assessment will be essential to make polycotton recycling economically and environmentally viable. As the textile industry moves toward circularity, these innovative strategies could significantly reduce the environmental footprint of blended fabrics and contribute to a more sustainable production system.

## Funding

NODES MUR – M4C2 1.5 of PNRR, European Union ‐ NextGenerationEU, (ECS00000036 – CUP, J83B22000050001).

## Conflicts of Interest

The authors declare no conflicts of interest.
